# Development and validation of a machine learning model for predicting preoperative deep vein thrombosis in elderly hip fracture patients

**DOI:** 10.3389/fmed.2026.1696325

**Published:** 2026-02-05

**Authors:** Xiaokang Wei, Zehao Yin, Shuqi Zhang, Maosheng Zhang, Dong Zhu

**Affiliations:** Department of Orthopedics, The First Hospital of Jilin University, Changchun, China

**Keywords:** deep vein thrombosis, hip fracture, machine learning, predictive model, SHAP value

## Abstract

**Background:**

Hip fractures in older adults pose a global health challenge. Deep vein thrombosis (DVT) is common, increasing surgical risk, delaying procedures, and causing severe thromboembolic events. It hampers recovery and lowers the quality of life. Prompt risk assessment and intervention are crucial. This study aims to develop and validate a machine learning model to predict DVT before surgery in elderly hip fracture patients, aiming to improve preoperative assessments and streamline clinical care pathways.

**Materials and methods:**

This study employed a retrospective design and included elderly patients who were hospitalized for hip fractures at a university-affiliated hospital between July 2022 and May 2025. A total of 782 patients met the inclusion criteria. The dataset was randomly divided into a training set (70%) and a validation set (30%). Five supervised machine learning algorithms were used to develop predictive models: decision tree (DT), extreme gradient boosting (XGBoost), support vector machine (SVM), light gradient boosting machine (LightGBM), and logistic regression (LR). Model performance was evaluated on the basis of discrimination, calibration, and clinical applicability, with SHAP analysis used for interpretability.

**Results:**

Among the 782 elderly patients with hip fractures, 186 (23.8%) DVT. Five features were selected for model construction: injury-to-admission time, D-dimer levels, hemoglobin levels, albumin levels, and activated partial thromboplastin time (APTT). Among all models, XGBoost achieved superior predictive accuracy, yielding an area under the receiver operating characteristic curve (AUC) of 0.829 (95% CI: 0.788–0.870) on the training set and 0.808 (95% CI: 0.742–0.874) on the validation set. Calibration curve assessment validated the model’s strong agreement between predicted and observed outcomes, and decision curve analysis (DCA) demonstrated notable clinical advantages.

**Conclusion:**

The XGBoost-based predictive model for preoperative DVT in elderly patients with hip fractures demonstrated superior performance. By integrating the SHAP method to enhance model interpretability and developing an intuitive web-based tool, the model’s clinical applicability was markedly improved. This predictive tool holds promise for assisting clinicians in risk assessment and guiding medical decision-making.

## 1 Introduction

As the global population ages rapidly, the incidence of hip fractures among older adults is rising, presenting a growing public health challenge worldwide (1). In 2019, an estimated 14.2 million hip fracture cases occurred worldwide, corresponding to an age-standardized incidence of 182 per 100,000 population (2). Hip fractures place a substantial burden on older adults, contributing to higher mortality, greater healthcare and societal costs, and marked declines in mobility and overall quality of life (3). For most older adults with hip fractures, surgical treatment remains the standard of care to achieve favorable outcomes; however, surgery carries inherent risks and potential complications (4).

Deep vein thrombosis (DVT) is a common complication in older adults with hip fractures, and its incidence increases markedly following delayed surgery or during prolonged immobilization (5). Older adults with hip fractures commonly have reduced mobility, physiological stress due to trauma, and impaired venous return, which together increase the risk of venous thrombus formation (6). These thrombi can obstruct venous flow and provoke local inflammation, leading to pain and swelling in the affected limb (7). Moreover, if a thrombus embolizes to the pulmonary circulation, it can cause a life-threatening pulmonary embolism (PE). The risk of venous thromboembolism after hip fracture is substantially elevated, with reported incidence rates for DVT and PE ranging from approximately 11 to 57% in this population, depending on study methodology and screening intensity (8–10). Consequently, timely identification and risk stratification of high-risk patients are essential to guide prophylactic strategies, reduce complications, and optimize perioperative management.

Conventional DVT risk assessment tools in orthopedics have limitations: many are derived from generalized risk factors, have not been validated specifically in orthopedic populations, and often rely on resource-intensive, operator-dependent imaging such as Doppler ultrasonography. Diagnostic delays arising from limited imaging availability or positioning constraints for patients with fractures can prolong preoperative waiting times and are associated with increased morbidity and mortality. Therefore, there is an urgent need for predictive approaches that are accurate, interpretable, and feasible to implement using routinely collected clinical data.

In recent years, machine learning techniques have increasingly been applied to predict postoperative or perioperative DVT in surgical populations. These studies underscore the growing clinical relevance of machine learning for DVT risk stratification in surgical and orthopedic settings (11, 12). Recent advances in artificial intelligence (AI) have rapidly expanded applications in biomedicine, offering novel approaches to address complex clinical problems (13). Machine learning (ML) models have demonstrated strong predictive performance across a broad spectrum of diseases and clinical outcomes (14). Interpretability methods, such as SHAP, provide actionable insights into model behavior, enhancing transparency and facilitating clinical translation (15). In this retrospective study, we developed and validated an interpretable machine-learning model to predict preoperative DVT risk in older adults with hip fractures, providing a clinically applicable tool for individualized risk stratification. Using routinely collected preoperative clinical and laboratory variables, we developed several predictive algorithms and found that Extreme Gradient Boosting (XGBoost) showed the best performance. To improve transparency, we used SHAP for model interpretability and deployed the final model as a web-based calculator to support real-time, individualized risk assessment within routine clinical workflows.

## 2 Materials and methods

### 2.1 Study design and patients

We conducted a retrospective observational study of elderly patients who sustained hip fractures and were admitted to a university-affiliated hospital between July 2022 and May 2025. Comprehensive clinical data were systematically extracted from the hospital’s electronic medical record system for subsequent analysis. The inclusion criteria were as follows: (1) patients aged 60 years or older; (2) radiographically confirmed hip fracture; and (3) completion of preoperative lower limb venous ultrasonography. The exclusion criteria included: (1) presence of multiple fractures, old fractures, or pathological fractures (e.g., resulting from bone metastases); (2) comorbidities potentially affecting DVT risk, such as hematological disorders, autoimmune diseases, chronic kidney disease, or liver cirrhosis, which may impact coagulation or hemodynamics; (3) a history of lower extremity venous thromboembolism diagnosed within 3 months prior to the fracture; (4) prior use of anticoagulant therapy (including rivaroxaban or low molecular weight heparin) before hospital admission; and (5) incomplete clinical data or failure to meet other study eligibility criteria. All elderly hip fracture patients admitted to our institution were managed with standardized DVT prophylaxis protocols, consistent with current clinical guidelines. Prophylactic strategies encompassed both mechanical and pharmacological modalities. Mechanical prophylaxis included the application of graduated compression stockings and intermittent pneumatic compression devices to enhance venous return in the lower extremities and reduce the risk of thrombosis. The pharmacological prophylaxis protocol involved subcutaneous administration of low molecular weight heparin (4,000 Axa IU/0.4 mL) or fondaparinux (2.5 mg) once daily. Patients who were diagnosed with DVT subsequently received anticoagulant and thrombolytic therapy (16). Preoperative venous ultrasonography was routinely conducted prior to anticoagulant administration, thereby avoiding potential interference of anticoagulation with DVT detection and related laboratory parameters.

### 2.2 Definition of lower extremity venous thrombosis ultrasonography

Lower extremity venous thrombosis, commonly referred to as DVT, is typically diagnosed using duplex ultrasonography, which combines two modalities: B-mode ultrasonography and Doppler flow assessment. The following diagnostic criteria are used: (1) Loss of Compressibility: The primary and most reliable sign of DVT is the inability to fully compress the vein with gentle transducer pressure. In normal veins, complete collapse is expected. A non-compressible vein indicates the presence of an intraluminal thrombus. (2) Intraluminal echogenic material: visualization of echogenic or hypoechoic thrombus within the lumen of the vein, which may partially or completely obstruct blood flow. (3) Absence or reduction of Doppler flow signal: In color Doppler imaging, diminished or absent flow signals within the affected vein segment suggest impaired venous blood flow due to thrombosis. (4) Lack of phasicity or augmentation: Spectral Doppler may show loss of normal respiratory variation (phasicity) or failure to augment flow with distal limb compression, both of which are consistent with proximal obstruction. Ultrasonography evaluation is most commonly performed on the common femoral, superficial femoral, popliteal, posterior tibial, and peroneal veins. A diagnosis of DVT is generally confirmed when one or more of the above features are present in the deep venous system of the lower extremities. All ultrasonographic assessments were conducted by experienced sonographers, each with at least a decade of clinical expertise, employing color Doppler ultrasonography and advanced imaging techniques to maximize diagnostic precision. All sonographic findings underwent independent review by a second qualified physician. If diagnostic ambiguity persisted, repeat ultrasonography examinations were performed to ensure diagnostic accuracy.

### 2.3 Data collection

Variables extracted for this study comprised baseline demographic and clinical information available within 24 h of admission. To protect patient confidentiality, all records were de-identified prior to analysis. The collected information included demographic characteristics such as gender, age, mechanism of injury, fracture site, injury-to-admission time (hours), as well as medical history, including hypertension, diabetes, cerebral infarction, surgical history, alcohol use history, and smoking history. Laboratory assessments encompassed white blood cell count (WBC), hemoglobin (HGB), mean corpuscular hemoglobin concentration (MCHC), mean corpuscular volume (MCV), platelet count (PLT), neutrophil count (NC), lymphocyte count (LYM), monocyte count (MONO), albumin (ALB), aspartate aminotransferase (AST), alanine aminotransferase (ALT), gamma-glutamyl transferase (GGT), total bilirubin (TBIL), creatinine (Cr), blood urea nitrogen (BUN), D-dimer, thrombin time (TT), activated partial thromboplastin time (APTT), prothrombin time (PT), and fibrinogen (FBG). Data extraction was performed independently by one author and subsequently validated for accuracy by a second independent reviewer.

### 2.4 Model construction and evaluation

Patients were randomly allocated to the training and validation sets in a 7:3 ratio. All data preprocessing procedures, including missing data imputation, feature selection, variable scaling, and hyperparameter tuning, were performed exclusively within the training set. The parameters derived from the training set were subsequently applied to the validation set without re-estimation. The validation set was used only for final model performance evaluation. In the training set, initial predictor selection was performed using least absolute shrinkage and selection operator (LASSO) regression implemented in the glmnet package in R. Predictors retained by LASSO were subsequently entered into a logistic regression model to estimate adjusted associations and to control for potential confounding. The final predictor set identified through the logistic regression was then used to develop a suite of machine-learning algorithms, including logistic regression (LR), a decision tree (DT), support vector machine (SVM), Light Gradient Boosting Machine (LightGBM), and Extreme Gradient Boosting (XGBoost). These algorithms were selected because of their strong performance on structured clinical data; their complementary modeling mechanisms and differing approaches to feature handling were expected to increase the diversity and robustness of model performance. Model discrimination was primarily assessed by the area under the receiver operating characteristic (ROC) curve (AUC). To provide a more complete assessment of classification performance, we also calculated accuracy, sensitivity (recall), and the F1 score. Differences in AUC between models were assessed using DeLong’s test for correlated ROC curves. Bootstrap-based internal validation (500 resamples) was applied to evaluate model discrimination and calibration. After selecting the optimal model, we applied SHAP analyses, including summary, dependence, and force plots, to examine model behavior at both global and individual (local) levels and thereby enhance interpretability.

### 2.5 Statistical analysis

Data analysis was performed using R software (version 4.4.2). Missing data were addressed using multiple imputation by chained equations (MICE), implemented with the ‘mice’ package (17). Multiple imputation using predictive mean matching (PMM) was performed with five imputations under a fixed random seed (123). The resulting imputed datasets were evaluated using the Akaike Information Criterion (AIC) and the Bayesian Information Criterion (BIC), and the imputed dataset with the lowest AIC and BIC values was selected for subsequent analyses (18) (Supplementary Tables 1–3). Continuous variables were reported as mean ± standard deviation (SD) for normally distributed data and as median (interquartile range) for non-normal distributions. Between-group comparisons for continuous data were performed using the independent samples *T*-test or the Mann–Whitney U test, as appropriate. Categorical variables were described as frequencies and percentages, and compared using the chi-square test or Fisher’s exact test when indicated.

### 2.6 Ethical considerations

All experimental procedures involving human participants strictly adhered to the ethical standards outlined in the 1964 Declaration of Helsinki and its subsequent revisions or equivalent ethical guidelines. This study, including the collection of electronic medical record information, was approved by the Ethics Committee of the First Hospital of Jilin University (Approval No: 2025-398).

## 3 Results

### 3.1 Patient demographics

**Figure 1:**
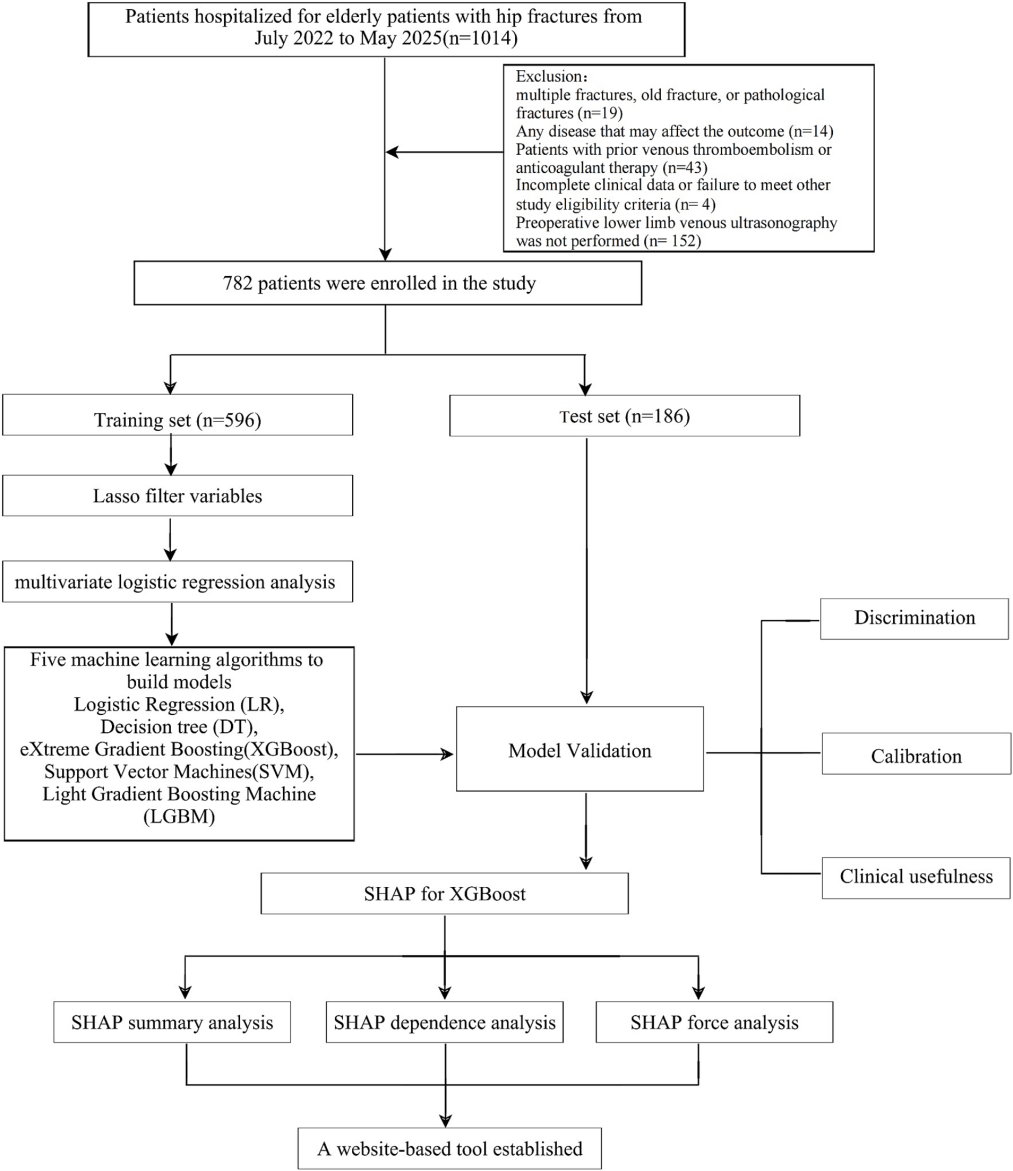
Flowchart of data screening and analysis.

A total of 1,014 elderly patients with hip fractures were initially screened for this study, of whom 782 met the predefined inclusion and exclusion criteria and were included in the analysis. The patient-selection process is illustrated in Figure 1. Baseline demographic and clinical characteristics for the training and validation cohorts are reported in Table 1, and the cohort distribution is summarized in Table 2. Comparisons of baseline characteristics between the training and validation cohorts showed no statistically significant differences (all *p* > 0.05), indicating that the two groups were well balanced prior to subsequent analyses.

**Table 1:** Comparison of the training set and validation set.

Variables	Validation set (*n* = 235)			Training set (*n* = 547)			*p* value
	No-DVT (*n* = 184)	DVT (*n* = 51)	*p* value	No-DVT (*n* = 412)	DVT (*n* = 135)	*p* value	
Gender, *N* (%)			1.000			0.010	0.031
Female	61 (33.15%)	17 (33.33%)		166 (40.29%)	37 (27.41%)		
Male	123 (66.85%)	34 (66.67%)		246 (59.71%)	98 (72.59%)		
Age, Median (Q1, Q3)	75.00 (67.00, 83.00)	80.00 (74.00, 86.00)	0.006	74.00 (68.00, 82.00)	78.00 (71.00, 84.00)	0.002	<0.001
Femoral neck fracture, *N* (%)			0.246			0.337	0.126
No	100 (54.35%)	33 (64.71%)		235 (57.04%)	84 (62.22%)		
Yes	84 (45.65%)	18 (35.29%)		177 (42.96%)	51 (37.78%)		
Mechanism of injury, *N* (%)			1.000			0.716	0.849
Low-energy trauma	175 (95.11%)	49 (96.08%)		390 (94.66%)	126 (93.33%)		
High-energy trauma	9 (4.89%)	2 (3.92%)		22 (5.34%)	9 (6.67%)		
Injury-to-admission time, Median (Q1, Q3)	8.50 (5.00, 24.00)	23.00 (7.00, 25.50)	0.022	9.50 (5.00, 24.00)	16.00 (5.50, 24.00)	0.015	0.001
Hypertension, *N* (%)			0.167			0.851	0.316
No	126 (68.48%)	29 (56.86%)		256 (62.14%)	82 (60.74%)		
Yes	58 (31.52%)	22 (43.14%)		156 (37.86%)	53 (39.26%)		
Diabetes, *N* (%)			0.988			0.348	0.329
No	154 (83.70%)	42 (82.35%)		329 (79.85%)	102 (75.56%)		
Yes	30 (16.30%)	9 (17.65%)		83 (20.15%)	33 (24.44%)		
Cerebral infarction, *N* (%)			0.025			0.029	0.002
No	52 (28.26%)	6 (11.76%)		114 (27.67%)	24 (17.78%)		
Yes	132 (71.74%)	45 (88.24%)		298 (72.33%)	111 (82.22%)		
Surgery history, *N* (%)			0.146			0.286	0.970
No	140 (76.09%)	33 (64.71%)		280 (67.96%)	99 (73.33%)		
Yes	44 (23.91%)	18 (35.29%)		132 (32.04%)	36 (26.67%)		
Alcohol use history, *N* (%)			1.000			0.696	0.751
No	180 (97.83%)	50 (98.04%)		406 (98.54%)	132 (97.78%)		
Yes	4 (2.17%)	1 (1.96%)		6 (1.46%)	3 (2.22%)		
Smoke history, *N* (%)			0.726			0.770	0.971
No	175 (95.11%)	48 (94.12%)		401 (97.33%)	131 (97.04%)		
Yes	9 (4.89%)	3 (5.88%)		11 (2.67%)	4 (2.96%)		
WBC, Median (Q1, Q3)	10.00 (8.05, 11.90)	9.33 (7.92, 11.96)	0.415	10.17 (8.22, 12.49)	9.82 (7.96, 12.26)	0.560	0.367
HGB, Median (Q1, Q3)	127.00 (111.00, 138.00)	119.00 (107.00, 127.00)	0.005	129.00 (117.00, 140.00)	122.00 (107.00, 133.00)	<0.001	<0.001
MCHC, Median (Q1, Q3)	336.00 (330.00, 343.00)	333.00 (324.50, 340.50)	0.028	335.00 (328.00, 343.00)	333.00 (327.00, 341.00)	0.238	0.027
MCV, Median (Q1, Q3)	91.40 (88.70, 95.43)	92.20 (89.80, 94.55)	0.656	91.55 (88.38, 94.80)	91.60 (88.35, 95.10)	0.926	0.778
PLT, Median (Q1, Q3)	192.50 (160.50, 234.25)	186.00 (153.00, 248.00)	0.866	195.00 (158.75, 232.00)	200.00 (162.00, 234.00)	0.442	0.574
NC, Median (Q1, Q3)	8.05 (6.41, 9.85)	7.22 (6.25, 10.13)	0.364	8.35 (6.30, 10.59)	7.82 (6.24, 10.62)	0.470	0.300
LYM, Median (Q1, Q3)	1.07 (0.83, 1.39)	1.08 (0.79, 1.40)	0.834	1.05 (0.78, 1.42)	1.09 (0.83, 1.37)	0.591	0.741
MONO, Median (Q1, Q3)	0.59 (0.46, 0.76)	0.59 (0.48, 0.73)	0.859	0.58 (0.46, 0.74)	0.60 (0.47, 0.78)	0.306	0.434
ALB, Median (Q1, Q3)	39.29 (35.16, 43.42)	37.32 (33.36, 41.28)	0.003	39.70 (37.08, 41.80)	38.20 (34.45, 40.90)	<0.001	<0.001
AST, Median (Q1, Q3)	23.30 (18.62, 28.92)	21.90 (17.25, 26.05)	0.032	22.05 (17.80, 28.50)	21.80 (18.35,27.30)	0.940	0.225
ALT, Median (Q1, Q3)	16.90 (12.20, 22.50)	14.80 (10.60, 20.10)	0.063	17.30 (12.80, 23.92)	15.40 (12.30, 20.15)	0.019	0.004
GGT, Median (Q1, Q3)	19.25 (14.40, 36.83)	16.40 (12.65, 24.50)	0.027	21.55 (15.40, 36.55)	17.80 (12.40, 37.00)	0.006	0.001
TBIL, Median (Q1, Q3)	14.10 (10.60, 18.22)	12.40 (9.95, 17.80)	0.381	13.60 (10.38, 18.50)	14.30 (9.55, 19.65)	0.679	0.924
CR, Median (Q1, Q3)	60.35 (47.25, 78.88)	63.10 (56.30, 80.00)	0.040	58.50 (49.70, 73.75)	60.00 (50.85, 70.90)	0.579	0.140
BUN, Median (Q1, Q3)	6.60 (5.28, 8.14)	7.69 (6.13, 10.19)	0.005	6.46 (5.19, 8.37)	6.59 (5.53, 8.16)	0.829	0.104
D-dimer, Median (Q1, Q3)	7.16 (3.47, 14.54)	15.02 (8.80, 25.42)	<0.001	6.91 (3.18, 15.52)	11.70 (6.34, 22.76)	<0.001	<0.001
TT, Median (Q1, Q3)	17.00 (16.30, 17.90)	16.80 (16.35, 17.65)	0.325	17.20 (16.50, 17.90)	17.10 (16.30, 18.00)	0.339	0.179
APTT, Median (Q1, Q3)	25.95 (24.40, 27.95)	25.40 (23.75, 27.15)	0.214	25.90 (24.50, 27.80)	25.30 (23.70, 27.05)	0.002	0.001
PT, Median (Q1, Q3)	11.60 (11.00, 12.10)	11.50 (11.10, 12.00)	0.889	11.60 (11.00, 12.12)	11.70 (11.10, 12.40)	0.357	0.454
FBG, Median (Q1, Q3)	3.06 (2.53, 3.86)	3.60 (2.88, 4.46)	0.004	3.10 (2.57, 3.80)	3.33 (2.53, 4.31)	0.065	0.002

WBC, white blood cell count; HGB, hemoglobin; MCHC, mean corpuscular hemoglobin concentration; MCV, mean corpuscular volume; PLT, platelet count; NC, neutrophil count; LYM, lymphocyte count; MONO, monocyte count; ALB, albumin; AST, alanine aminotransferase; ALT, alanine aminotransferase; GGT, gamma-glutamyl transferase; TBIL, total bilirubin; CR, creatinine; BUN, blood urea nitrogen; TT, thrombin time; APTT, activated partial thromboplastin time; PT, prothrombin time; FBG, fibrinogen; DVT, deep vein thrombosis.

**Table 2:** Baseline clinical characteristics in train cohort and test cohort.

Variables	Total (*n* = 782)	Validation set (*n* = 235)	Training set (*n* = 547)	*p* value
Gender, *N* (%)				0.334
Female	281 (35.93%)	78 (33.19%)	203 (37.11%)	
Male	501 (64.07%)	157 (66.81%)	344 (62.89%)	
Age, Median (Q1, Q3)	76.00 (68.00, 83.00)	76.00 (67.50, 84.00)	76.00 (68.00, 83.00)	0.526
Femoral neck fracture, *N* (%)				0.713
No	452 (57.80%)	133 (56.60%)	319 (58.32%)	
Yes	330 (42.20%)	102 (43.40%)	228 (41.68%)	
Mechanism of injury, *N* (%)				0.698
Low-energy trauma	740 (94.63%)	224 (95.32%)	516 (94.33%)	
High-energy trauma	42 (5.37%)	11 (4.68%)	31 (5.67%)	
Injury-to-admission time, Median (Q1, Q3)	10.00 (5.00, 24.00)	10.00 (5.00, 24.00)	10.00 (5.00, 24.00)	0.930
Hypertension, *N* (%)				0.305
No	493 (63.04%)	155 (65.96%)	338 (61.79%)	
Yes	289 (36.96%)	80 (34.04%)	209 (38.21%)	
Diabetes, *N* (%)				0.166
No	627 (80.18%)	196 (83.40%)	431 (78.79%)	
Yes	155 (19.82%)	39 (16.60%)	116 (21.21%)	
Cerebral infarction, *N* (%)				0.943
No	196 (25.06%)	58 (24.68%)	138 (25.23%)	
Yes	586 (74.94%)	177 (75.32%)	409 (74.77%)	
Surgery history, *N* (%)				0.257
No	552 (70.59%)	173 (73.62%)	379 (69.29%)	
Yes	230 (29.41%)	62 (26.38%)	168 (30.71%)	
Alcoholism history, *N* (%)				0.769
No	768 (98.21%)	230 (97.87%)	538 (98.35%)	
Yes	14 (1.79%)	5 (2.13%)	9 (1.65%)	
Smoke history, *N* (%)				0.148
No	755 (96.55%)	223 (94.89%)	532 (97.26%)	
Yes	27 (3.45%)	12 (5.11%)	15 (2.74%)	
WBC, Median (Q1, Q3)	10.07 (8.06, 12.33)	9.83 (7.99, 11.91)	10.12 (8.11, 12.48)	0.281
HGB, Median (Q1, Q3)	127.00 (112.00, 138.00)	125.00 (110.00, 137.00)	128.00 (114.00, 138.00)	0.086
MCHC, Median (Q1, Q3)	334.00 (328.00, 342.00)	335.00 (328.00, 342.00)	334.00 (328.00, 342.00)	0.749
MCV, Median (Q1, Q3)	91.50 (88.60, 95.07)	91.40 (89.00, 95.30)	91.60 (88.35, 94.90)	0.410
PLT, Median (Q1, Q3)	195.00 (159.00, 234.00)	191.00 (158.50, 236.50)	196.00 (159.00, 232.00)	0.900
NC, Median (Q1, Q3)	8.11 (6.29, 10.45)	7.93 (6.34, 10.03)	8.20 (6.29, 10.59)	0.215
LYM, Median (Q1, Q3)	1.06 (0.80, 1.40)	1.08 (0.82, 1.40)	1.06 (0.80, 1.41)	0.818
MONO, Median (Q1, Q3)	0.59 (0.46, 0.75)	0.59 (0.46, 0.75)	0.58 (0.46, 0.75)	0.796
TP, Median (Q1, Q3)	39.20 (36.00, 41.70)	39.10 (35.85, 41.90)	39.20 (36.20, 41.65)	0.840
AST, Median (Q1, Q3)	22.40 (17.92, 28.30)	22.80 (18.10, 28.75)	21.90 (17.90, 28.20)	0.258
ALT, Median (Q1, Q3)	16.70 (12.40, 22.80)	16.30 (11.90, 21.70)	16.80 (12.70, 23.05)	0.209
GGT, Median (Q1, Q3)	20.05 (14.30, 36.00)	18.60 (13.65, 34.70)	20.80 (14.50, 36.60)	0.092
TBIL, Median (Q1, Q3)	13.70 (10.30, 18.50)	13.80 (10.45, 18.10)	13.70 (10.25, 18.70)	0.938
CR, Median (Q1, Q3)	59.90 (49.70, 74.77)	61.30 (49.30, 80.00)	58.90 (49.75, 72.50)	0.131
BUN, Median (Q1, Q3)	6.58 (5.30, 8.40)	6.79 (5.50, 8.54)	6.48 (5.28, 8.32)	0.225
D-dimer, Median (Q1, Q3)	8.40 (3.71, 17.39)	8.90 (3.99, 15.92)	8.25 (3.57, 17.91)	0.462
TT, Median (Q1, Q3)	17.10 (16.40, 17.90)	17.00 (16.30, 17.90)	17.20 (16.40, 17.90)	0.211
APTT, Median (Q1, Q3)	25.75 (24.30, 27.60)	25.80 (24.25, 27.80)	25.70 (24.30, 27.50)	0.849
PT, Median (Q1, Q3)	11.60 (11.00,12.20)	11.60 (11.00, 12.10)	11.60 (11.00, 12.20)	0.520
FBG, Median (Q1, Q3)	3.15 (2.57, 3.95)	3.16 (2.64, 3.94)	3.14 (2.54, 3.96)	0.467

WBC, white blood cell count; HGB, hemoglobin; MCHC, mean corpuscular hemoglobin concentration; MCV, mean corpuscular volume; PLT, platelet count; NC, neutrophil count; LYM, lymphocyte count; MONO, monocyte count; ALB, albumin; AST, alanine aminotransferase; ALT, alanine aminotransferase; GGT, gamma-glutamyl transferase; TBIL, total bilirubin; CR, creatinine; BUN, blood urea nitrogen; TT, thrombin time; APTT, activated partial thromboplastin time; PT, prothrombin time; FBG, fibrinogen; DVT, deep vein thrombosis.

### 3.2 Variables selection

The occurrence of DVT was defined as the dependent outcome for this analysis. The LASSO regression was applied to the prespecified pool of candidate predictors to select the most relevant risk factors for preoperative DVT in elderly patients with hip fractures. The optimal penalty parameter (*λ*) for the LASSO model was selected from 31 candidate predictors using 10-fold cross-validation to balance model complexity and predictive performance. We adopted the lambda.1se rule, defined as the largest λ within one standard error of the minimum cross-validation error, which yields the simplest model whose error remains within the cross-validation error range. Compared with lambda.min (the λ that attains the minimum cross-validation error), the lambda.1se choice is more conservative and tends to improve model stability and generalizability (Supplementary Figure 1). Six significant variables were identified: gender, injury-to-admission time, hemoglobin levels, albumin levels, D-dimer levels, and APTT. These variables were subsequently incorporated into a logistic regression analysis to control for confounding factors. The final analysis demonstrated that injury-to-admission time, hemoglobin levels, albumin levels, APTT, and D-dimer levels were independent predictors of preoperative lower limb DVT (*p* < 0.05). Detailed results are presented in Table 3.

**Table 3:** Multivariate logistic regression analysis of the risk of DVT.

Characteristics	*β*	SE	Wald	OR	CI	*p* value
APTT (s)	−0.14	0.042	−3.341	0.869	0.869 (0.798–0.940)	<0.001
Injury-to-admission time (hours)	0.018	0.005	3.566	1.018	1.018 (1.008–1.028)	<0.001
Hemoglobin (g/L)	−0.015	0.007	−2.177	0.986	0.985 (0.972–0.998)	<0.05
D-dimer (μg/ml)	0.041	0.008	5.158	1.042	1.041 (1.026–1.058)	<0.001
albumin (g/L)	−0.096	0.032	−3.008	0.909	0.908 (0.853–0.966)	<0.01

APTT, activated partial thromboplastin time; SE, standard error; OR, odds ratio; CI, confidence interval.

### 3.3 Establishment and evaluation of the best model

The final set of predictive variables was used to train five supervised machine-learning algorithms: DT, LR, SVM, LightGBM, and XGBoost. Models were developed using the training dataset and were comprehensively evaluated for discrimination, calibration, and clinical utility. Discrimination refers to a model’s ability to distinguish between patients who develop DVT and those who do not. Calibration assesses agreement between predicted probabilities and observed outcomes. Clinical utility denotes the net benefit of applying the model in real-world clinical decision-making. The final hyperparameters for each model are reported in Supplementary Table S4.

Model performance was assessed using a suite of metrics, including the receiver operating characteristic (ROC) curve and its area under the curve (AUC), accuracy, sensitivity, specificity, precision, and F1 score. Detailed comparisons of discrimination metrics for all five models in both the training and validation datasets are presented in Table 4. DeLong’s test indicated that the AUC of the XGBoost model was significantly higher than those of the DT, LR, SVM, and LightGBM models. Model calibration was assessed using the Brier score and Spiegelhalter’s Z test. XGBoost yielded the lowest Brier score, suggesting better overall calibration. Furthermore, Spiegelhalter’s Z test did not indicate a significant lack of fit for the XGBoost model. Detailed statistical results for discrimination and calibration comparisons among all models are presented in Supplementary Table S5. Bootstrap-based internal validation (500 resamples) confirmed that XGBoost achieved the highest mean AUC and the lowest Brier score among the evaluated models (Supplementary Tables S6, S7), supporting its superior and stable predictive performance.

**Table 4:** Model discrimination assessment.

Dataset	Model	AUC	Accuracy	Sensitivity	Specificity	Precision	Recall	F1
Training	LR	0.731	0.655	0.733	0.629	0.393	0.733	0.512
	XGB	0.829	0.779	0.748	0.789	0.537	0.748	0.625
	SVM	0.709	0.600	0.800	0.534	0.360	0.800	0.497
	LightGBM	0.786	0.768	0.607	0.820	0.526	0.607	0.564
	DT	0.788	0.841	0.793	0.850	0.482	0.793	0.599
Validation	LR	0.749	0.647	0.706	0.630	0.346	0.706	0.465
	XGB	0.808	0.723	0.726	0.723	0.421	0.726	0.532
	SVM	0.743	0.583	0.784	0.527	0.315	0.784	0.449
	LightGBM	0.721	0.664	0.510	0.707	0.325	0.510	0.397
	DT	0.746	0.749	0.400	0.821	0.314	0.400	0.352

AUC, area under the curve; LR, logistic regression; SVM, support vector machine; LightGBM, light gradient boosting machine; XGBoost, extreme gradient boosting; DT, decision tree.

**Figure 2:**
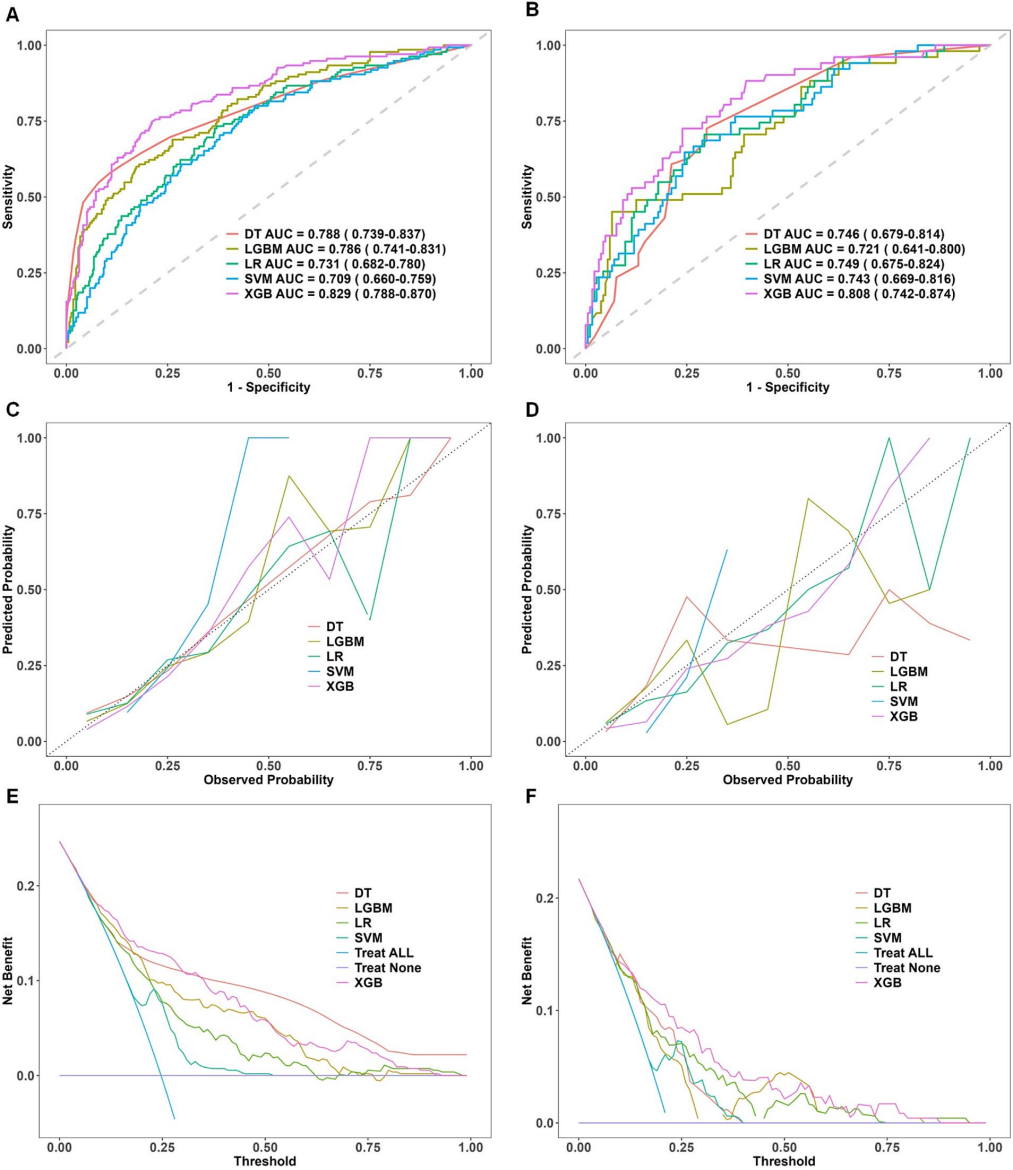
Comparative performance evaluation of machine learning models. Performance evaluation of five machine learning models—decision tree (DT), light gradient boosting machine (LGBM), logistic regression (LR), support vector machine (SVM), and extreme gradient boosting (XGBoost)—in the training and validation datasets. **(A,B)** ROC curves with AUC values, showing XGBoost’s superior discriminative performance. **(C,D)** Calibration curves indicating a strong fit and robust generalization for XGBoost. **(E,F)** DCA curves demonstrating XGBoost’s net clinical benefit. ROC, receiver operating characteristic; DCA, decision curve analysis; AUC, area under the curve; DT, decision tree; LR, logistic regression; SVM, support vector machine; LGBM, light gradient boosting machine; XGBoost, extreme gradient boosting. Panels **(A,C,E)** represent results from the training dataset, whereas Panels **(B,D,F)** correspond to results from the validation dataset.

Among the models tested, XGBoost achieved the best discriminative performance: AUC = 0.829 (95% CI, 0.788–0.870) in the training set and AUC = 0.808 (95% CI, 0.742–0.874) in the validation set. The corresponding ROC curves are shown in Figures 2A,B.

Calibration curves were constructed to compare observed incidences of preoperative DVT with model-predicted probabilities in the training and validation cohorts. The XGBoost calibration curve was closest to the ideal calibration line, indicating greater concordance between predicted and observed probabilities. This finding underscores the model’s robustness in estimating clinically realistic probabilities. The corresponding calibration plots are shown in Figures 2C,D.

Decision curve analysis was used to assess clinical utility. Compared with other algorithms, XGBoost demonstrated substantial net benefit across a wide range of threshold probabilities in both cohorts. In the validation cohort, the decision curve for XGBoost remained consistently above the default strategies of treating all or treating none for threshold probabilities between 0.05 and 0.90, indicating superior clinical utility. The decision curves are shown in Figures 2E,F.

Taken together, these results identify XGBoost as the best-performing predictive model for this dataset, demonstrating strong discrimination, good calibration, and favorable decision-curve performance. These properties support its potential utility for individualized clinical decision-making.

### 3.4 Model interpretation and clinical significance analysis

To further clarify the clinical utility of the XGBoost model, we applied SHAP to interpret the model’s prediction mechanisms and outputs. SHAP enables the quantification and visualization of each feature’s contribution to model predictions, thereby enhancing transparency and facilitating clinical integration. Interpretability was examined at two levels: a global perspective, which characterizes overall feature importance and model behavior, and a local perspective, which provides predictions for individual patients.

**Figure 3:**
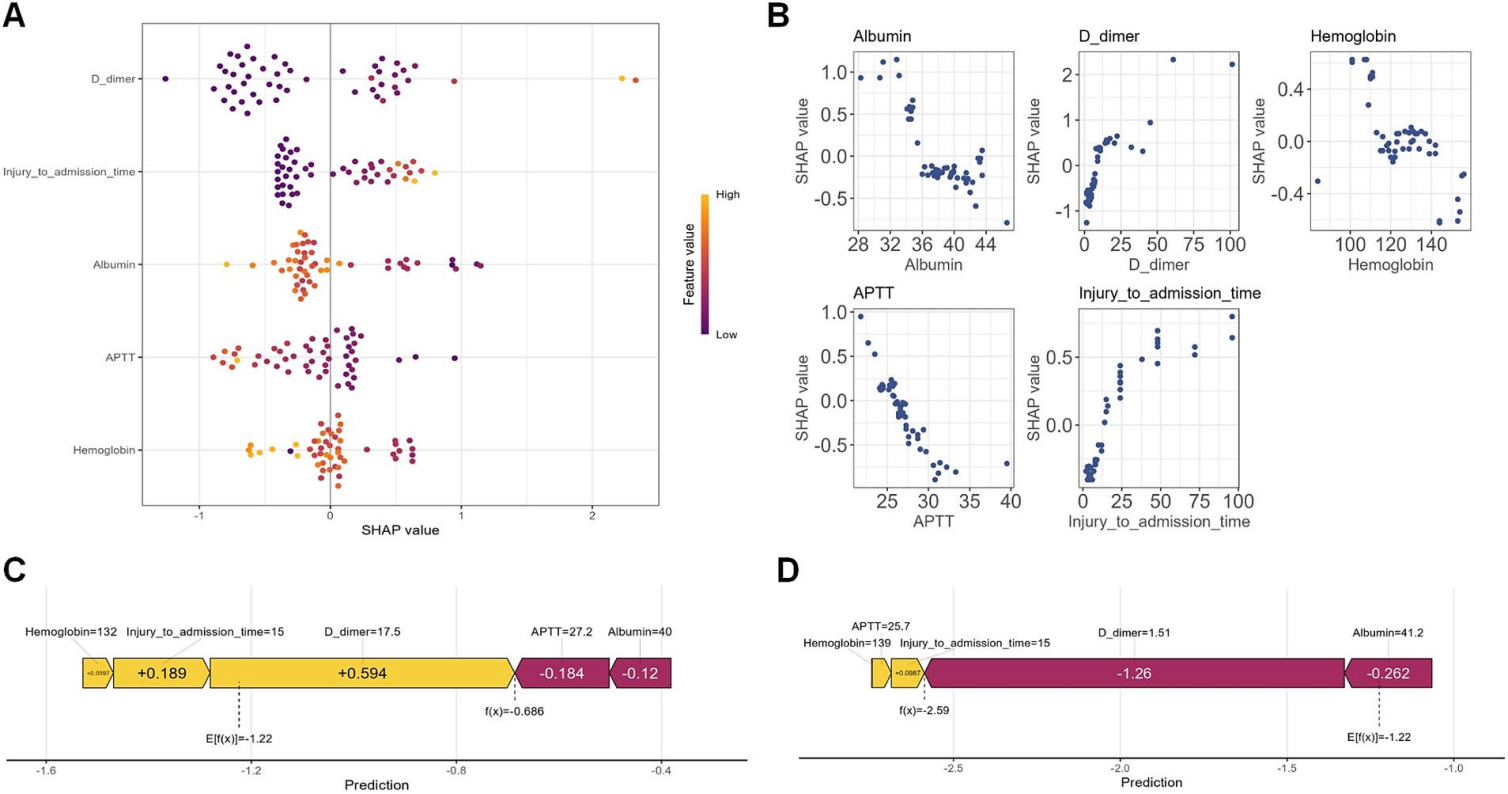
**(A)** SHAP feature importance plot. The SHAP summary plot ranks feature importance in the XGBoost model based on mean SHAP values. Feature values are indicated by a color gradient, with yellow representing high values and purple representing low values. **(B)** SHAP dependence plot. The SHAP dependence plots illustrate the marginal effects of five key features on the model’s predictions, highlighting the relationships between deep vein thrombosis (DVT) and its predictive factors. **(C)** SHAP force plot for a true positive case. The SHAP force plot for a true positive patient shows a SHAP value of −0.686, exceeding the model’s baseline (E[f(x)] = −1.22). Positive contributions from Hemoglobin, Injury-to-admission time, D-dimer, and (in yellow) increased the predicted likelihood of DVT, while Albumin and APTT (in red) exerted a negative influence, reducing the predicted risk. **(D)** SHAP force plot for a true negative case. In contrast, the SHAP force plot for a true negative patient yielded a SHAP value of −2.59, below the baseline. Negative contributions from D-dimer and Albumin (in red) reduced the predicted probability of DVT, whereas minor positive contributions from Hemoglobin, APTT, and Injury-to-admission time (in yellow) had a limited effect.

Figure 3A shows the SHAP summary (beeswarm) plot, illustrating the relative importance and impact of each predictor on the model’s estimated preoperative risk of deep vein thrombosis in elderly patients with hip fractures. Each point represents a single patient’s SHAP value for a given variable, indicating the direction and magnitude of that variable’s effect on the prediction. Features are ordered on the y-axis by their mean absolute SHAP values. The x-axis indicates direction: positive SHAP values increase predicted risk, while negative SHAP values decrease predicted risk. In our model, the top predictors in descending order of influence were D-dimer, time from injury to admission, serum albumin, activated partial thromboplastin time (APTT), and hemoglobin.

Figure 3B shows the SHAP dependence plots for the five key predictors. The horizontal axis displays the raw feature values, and the vertical axis presents the corresponding SHAP scores, which indicate the magnitude and direction of each feature’s contribution to predicted DVT risk. Points with positive SHAP values (above zero) denote increased predicted risk, while points with negative SHAP values (below zero) denote decreased risk. Thus, a positive SHAP value corresponds to a higher likelihood of preoperative DVT, whereas a negative value corresponds to a lower likelihood. This visualization enables clinicians to assess directly how changes in each variable influence individual model predictions.

Compared with traditional “black-box” models, SHAP force plots provide clear, case-specific explanations for individual predictions by quantifying each feature’s impact. Rooted in cooperative game theory, SHAP assigns each variable an importance score that reflects its marginal contribution to the model’s output. In the XGBoost model, the baseline SHAP value, E[f(x)] = −1.22, serves as the reference for interpreting patient-specific risk. Features that increase risk are shown as yellow arrows pointing right, while features that decrease risk are shown as red arrows pointing left. For example, Figure 3C illustrates a true positive with a SHAP value of −0.686, indicating an increased predicted DVT risk relative to baseline. By contrast, Figure 3D shows a true negative with a SHAP value of −2.59, corresponding to a lower predicted risk. By providing case-specific explanations, SHAP enhances model transparency compared with conventional approaches and supports personalized, data-driven clinical decision-making.

### 3.5 Risk prediction applet

**Figure 4:**
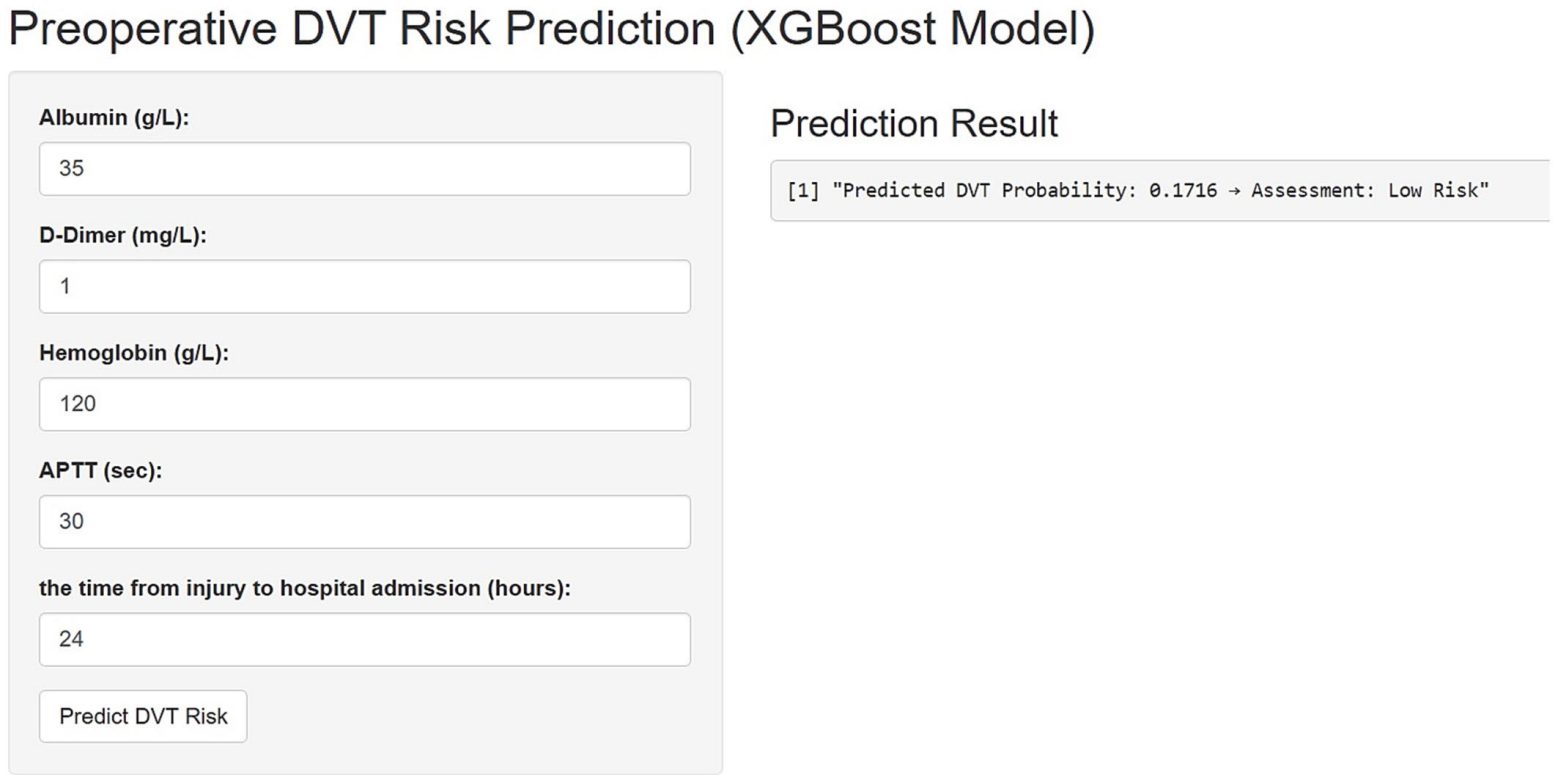
An online platform has been developed to predict the risk of preoperative deep vein thrombosis (DVT) in elderly patients with hip fractures. The web-based tool, accessible via https://3123213wxk.shinyapps.io/1111/, enables clinicians to perform real-time risk assessments by entering individual patient data. Through an interactive visual interface, the application estimates the likelihood of DVT occurrence based on key clinical characteristics.

Figure 4 illustrates the implementation of the finalized predictive model as an interactive web-based tool, optimized for integration into clinical workflows. By entering patient-specific values for the five key predictors, the application automatically computes the estimated probability of preoperative DVT in elderly hip fracture patients, thereby facilitating real-time risk assessment and supporting informed clinical decision-making.

## 4 Discussion

In this study, we developed an XGBoost model interpretable via SHAP using five routinely collected preoperative clinical variables to predict the risk of DVT in elderly patients with hip fractures. Compared with conventional risk assessment tools, our approach not only achieved strong predictive performance but also provided transparent patient-level explanations of risk. We used LASSO followed by multivariable regression for rigorous predictor selection and identified injury-to-admission time, D-dimer, hemoglobin, albumin, and APTT as the most informative features. SHAP-based interpretability helps clinicians visualize feature-level contributions to individual predictions and supports more personalized thromboprophylaxis decisions in this high-risk population. Nevertheless, external validation and prospective impact studies are required to confirm generalizability and clinical utility before routine implementation.

Among elderly adults with hip fractures, injury-to-admission time may play a pivotal role in the onset of preoperative DVT. Prolonged delays in this interval frequently lead to extended immobility and compromised venous circulation, both of which are key components of Virchow’s triad and recognized risk factors for thrombus formation (19, 20). This susceptibility is further exacerbated in the elderly owing to age-related vascular alterations and an inherently elevated baseline risk of coagulation disorders (21, 22). Previous studies have consistently demonstrated an association between delayed hospital admission and a higher incidence of DVT, highlighting the importance of prompt risk identification and expedited patient transfer to medical facilities (23). Optimizing early triage and reducing pre-hospital wait times could therefore be critical in lowering thrombotic complications. Our observations support the view that injury-to-admission time might serve as an early clinical signal to flag patients at elevated risk of DVT.

We found that a shorter APTT can hint at hidden clotting risk in elderly hip-fracture patients (24). Following trauma, tissue injury leads to increased thrombin production via activation of both intrinsic and extrinsic coagulation pathways; as thrombin levels rise, APTT values decrease (25). This reduction in APTT, which may go unnoticed in the absence of overt bleeding or coagulation disorders, is often associated with increased coagulation activity and reduced fibrinolysis, creating a favorable environment for venous thrombosis (26). Due to age-related declines in vascular and hemostatic regulatory capacity, even subtle changes in coagulation parameters have greater clinical significance in elderly individuals compared to younger patients. Adding APTT to the routine preoperative checklist is inexpensive and quick, and it may help flag patients who need closer surveillance or early anticoagulation, even though it works best alongside other risk markers.

Low albumin levels turned out to be an independent warning sign for preoperative DVT in our older hip-fracture cohort. Albumin is the most abundant protein in plasma, maintaining intravascular oncotic pressure, stabilizing the vascular endothelium, and modulating both inflammatory and coagulation processes (27–29). When albumin levels decrease, it often reflects not only malnutrition but also increased systemic inflammation and endothelial dysfunction, which together promote a hypercoagulable state. Two main pathways explain why low albumin tilts the balance toward clotting. First, with less oncotic pull, fluid seeps into tissues and slows venous flow, giving clots time to form (30). Second, Albumin normally binds and neutralizes pro-thrombotic molecules such as platelet-activating factors and free fatty acids. A deficiency of albumin allows these pro-thrombotic factors to accumulate, thereby enhancing platelet aggregation and activation of the coagulation cascade (31–33). Multiple clinical studies have shown that hypoalbuminemia is associated with increased thrombotic risk in surgical and hospitalized patients. Preoperative albumin assessment is a simple and cost-effective way to identify individuals who may require more intensive DVT prophylaxis, and it encourages clinicians to consider both nutritional status and inflammation in the management of elderly fracture patients.

We observed that older hip-fracture patients with lower hemoglobin levels faced a noticeably higher chance of preoperative DVT. Hemoglobin is best known for carrying oxygen, yet falling levels seem to nudge the clotting system as well (34). When hemoglobin drops, tissues slip into mild hypoxia. That oxygen shortfall ramps up HIF-1α, boosts tissue-factor release, and activates the endothelium changes that spark platelet clumping, accelerate the coagulation cascade, and blunt fibrinolysis, shifting the blood toward a hyper-coagulable state (35, 36). In elderly patients, anemia is frequently associated with chronic inflammation, malnutrition, and multiple comorbidities. Inflammatory cytokines can both slow red-cell production and kickstart clotting pathways, adding to the risk. Considering anemia solely as a disorder of oxygen transport overlooks its broader role as an indicator of increased thrombotic risk. Flagging low hemoglobin early lets clinicians fine-tune preoperative plans and start thromboprophylaxis sooner for these fragile, high-risk patients.

In our study, elevated D-dimer levels were closely associated with preoperative deep vein thrombosis. Although D-dimer is simply a fragment of broken-down fibrin, rising levels signal that clots are being made and dismantled at the same time (37, 38). A fracture sets that cycle in motion: tissue damage, less movement, and other preoperative stresses switch on both intrinsic and extrinsic clotting routes, driving fibrin build-up. As those fresh clots start to dissolve, D-dimer drifts into the bloodstream, an early chemical hint that thrombosis is active (39). Because of this quick “on–off” feedback, D-dimer has become a handy but broad screening tool, especially in older trauma patients who sit on a higher baseline risk (40). A sharp rise can justify early imaging or pre-emptive anticoagulation, even before clinical signs surface. D-dimer alone cannot seal a diagnosis, yet folding it into the preoperative work-up offers real value. It provides clinicians with timely information regarding coagulation system activity, which is particularly important for the management of vulnerable, high-risk patients.

During model development, we systematically compared multiple machine-learning algorithms, including multivariate logistic regression, decision trees, LightGBM, and XGBoost, to identify the most robust predictive model for preoperative DVT risk. Our findings demonstrated that XGBoost, in particular, excelled at capturing nonlinear and high-dimensional interactions, resulting in superior predictive accuracy and generalizability. Rigorous cross-validation and diverse performance metrics (AUC, accuracy, sensitivity, specificity) confirmed the reliability and clinical relevance of our results, with XGBoost consistently outperforming other models—especially in identifying high-risk patients requiring timely intervention. Beyond predictive power, the integration of SHAP analysis allowed us to visualize both global and individualized feature contributions, thereby enhancing model transparency. This level of interpretability is essential for clinician trust and widespread adoption of AI-driven tools in clinical practice. Taken together, our results highlight that an interpretable XGBoost-based model, combined with intuitive SHAP explanations, offers substantial promise for improving DVT risk stratification and guiding personalized prophylactic strategies in geriatric orthopedics.

Venography remains the gold standard for diagnosing DVT, but its invasiveness and radiation exposure restrict routine clinical use. Color Doppler ultrasonography is widely employed, yet its accuracy depends on operator expertise, and it is difficult to deploy in primary care, particularly for patients with lower limb fractures, because of positioning constraints. Moreover, waiting for these tests can delay surgery and adversely affect outcomes. Clinical guidelines advocate early surgical management for hip fracture patients because extended preoperative delays increase the risk of complications. To address these barriers, we developed an XGBoost-based machine learning model that uses clinical history and routine laboratory tests to rapidly identify patients at high risk for DVT. This approach can prompt timely specialist assessment, reduce financial burden, shorten waiting time, and ultimately contribute to improved patient prognosis.

This study has several limitations. First, its retrospective single-center design, based on electronic medical records, may have introduced selection and information bias; certain key variables, such as body mass index, genetic thrombophilia, or detailed medication history, were unavailable, potentially leading to unmeasured confounding. Second, although the model was rigorously developed and internally validated, its generalizability remains uncertain without external validation in independent cohorts or diverse populations. Third, our analysis focused exclusively on preoperative DVT risk and did not capture the impact of postoperative complications or long-term outcomes, which may further influence patient prognosis. Fourth, while multiple imputation (MICE) addressed missing data, this technique cannot entirely eliminate bias from non-random or extensive data gaps. Fifth, compared with large multicenter cohorts, the sample is relatively small, which may constrain performance stability and limit generalizability across settings. Lastly, variations in perioperative management and local clinical protocols may have affected DVT risk but were not systematically evaluated.

Future research should prioritize multicenter prospective validation, inclusion of broader clinical variables, and integration of postoperative and long-term follow-up data to further enhance model performance and clinical utility.

## 5 Conclusion

In this retrospective cohort of geriatric patients with hip fractures, we identified five independent preoperative predictors of DVT: time from injury to admission, hemoglobin, albumin, D-dimer, and APTT. Using these variables, we developed and internally validated five machine learning models; the XGBoost model showed the best discrimination and calibration in the validation set. *Post hoc* SHAP analysis provided case-level explanations of feature contributions, and we deployed the final model through a web browser interface to deliver clinicians real-time, interpretable risk estimates that can inform targeted thromboprophylaxis and preoperative decision making.

## Data Availability

The raw data supporting the conclusions of this article will be made available by the authors, without undue reservation.
