# Supplementary material for: Development and validation of a machine learning model for predicting preoperative deep vein thrombosis in elderly hip fracture patients
Source: Front Med (Lausanne). 2026 Feb 5;13:1696325. doi: 10.3389/fmed.2026.1696325 (PMC12916597; doi:10.3389/fmed.2026.1696325)
Supplement: Supplementary file 1 [file Data_Sheet_1.docx]

**Supplementary Material**

**1. Supplementary Tables and Figures**

Table S1. Assessment of multiple imputation (MICE) performance.

| model | Null deviance | Df null | LogLik | AIC | BIC | Deviance | Df residual | Nobs |
| --- | --- | --- | --- | --- | --- | --- | --- | --- |
| 1 | 858 | 781 | -382 | 791 | 851 | 765 | 769 | 782 |
| 2 | 858 | 781 | -383 | 792 | 853 | 766 | 769 | 782 |
| 3 | 858 | 781 | -380 | 787 | 847 | 761 | 769 | 782 |
| 4 | 858 | 781 | -378 | 783 | 843 | 757 | 769 | 782 |
| 5 | 858 | 781 | -383 | 793 | 853 | 767 | 769 | 782 |

The table reports fit statistics for five candidate models (n = 782). Model 4 exhibited the best in-sample fit (highest logLik = -378, lowest AIC = 783 and BIC = 843, and lowest residual deviance = 757). Df null, degrees of freedom for null model; LogLik, log-likelihood; AIC, Akaike Information Criterion; BIC, Bayesian Information Criterion; Df residual, residual degrees of freedom; Nobs, number of observations

Table S2. Variables with missing values and their missing rates.

| Variable | Missing rate, n (%) |
| --- | --- |
| D-dimer | 143 (18.3%) |
| ALB | 2 (0.3%) |
| AST | 2 (0.3%) |
| ALT | 2 (0.3%) |
| GGT | 2 (0.3%) |
| TBIL | 2 (0.3%) |
| CR | 1 (0.1%) |
| BUN | 1 (0.1%) |
| TT | 3 (0.4%) |
| APTT | 3 (0.4%) |
| PT | 3 (0.4%) |
| FBG | 3 (0.4%) |

The table lists variables with missing values and their respective missing rates before imputation. No key predictor exceeded 20% missingness. ALB, albumin; AST, alanine aminotransferase; ALT, alanine aminotransferase; GGT, gamma-glutamyl transferase; TBIL, total bilirubin; CR, creatinine; BUN, blood urea nitrogen; TT, thrombin time; APTT, activated partial thromboplastin time; PT, prothrombin time; FBG, fibrinogen.

Table S3. Comparisons of the data before and after imputation.

| Variable | Before imputation (n=782) | After imputation (n=782) | *p* value |
| --- | --- | --- | --- |
| D-dimer | 8.68 (3.56, 18.09) | 8.40 (3.71, 17.39) | 0.533 |
| ALB | 39.20 (36.00, 41.70) | 39.20 (36.00, 41.70) | 0.977 |
| AST | 22.40 (17.98, 28.35) | 22.40 (17.92, 28.30) | 0.950 |
| ALT | 16.70 (12.40, 22.83) | 16.70 (12.40, 22.80) | 0.962 |
| GGT | 20.05 (14.30, 36.00) | 20.05 (14.30, 36.00) | 0.977 |
| TBIL | 13.70 (10.30, 18.50) | 13.70 (10.30, 18.50) | 0.965 |
| CR | 59.90 (49.70, 74.80) | 59.90 (49.70, 74.77) | 0.989 |
| BUN | 6.58 (5.30, 8.40) | 6.58 (5.30, 8.40) | 0.977 |
| TT | 17.10 (16.40, 17.90) | 17.10 (16.40, 17.90) | 0.942 |
| APTT | 25.70 (24.30, 27.60) | 25.75 (24.30, 27.60) | 0.969 |
| PT | 11.60 (11.00, 12.20) | 11.60 (11.00, 12.20) | 0.953 |
| FBG | 3.14 (2.57, 3.95) | 3.15 (2.57, 3.95) | 0.926 |

The table compares the distributions of variables before and after imputation using median (Q1, Q3) values. No significant differences were observed between the original and imputed datasets (all *p* > 0.05), confirming the stability of the imputation process. ALB, albumin; AST, alanine aminotransferase; ALT, alanine aminotransferase; GGT, gamma-glutamyl transferase; TBIL, total bilirubin; CR, creatinine; BUN, blood urea nitrogen; TT, thrombin time; APTT, activated partial thromboplastin time; PT, prothrombin time; FBG, fibrinogen.

Table S4. Final hyperparameters adopted in the five ML models

| **ML models** | **Hyperparameters** |
| --- | --- |
| XGboost | booster = "gbtree"  learning_rate = 0.3  n_estimators = 1000  max_depth = 2  gamma = 0.001  subsample = 0.7  colsample_bytree = 0.4  objective = "binary:logistic"  eval_metric = "logloss"  early_stopping_rounds = 200 |
| LightGBM | objective = "binary"  n_estimators = 981  n_features = 5 |
| SVM | kernel = "linear"  cost = 1 |
| DT | cp = 0.01 |
| LR | - |

XGBoost, extreme gradient boosting; LightGBM, light gradient boosting machine; SVM, support vector machine; DT, decision tree; LR, logistic regression.

All predictive models were implemented in R and trained exclusively on the training dataset to prevent information leakage. Hyperparameter optimization was performed within the training set using stratified cross-validation. Model selection was primarily based on the mean cross-validated area under the receiver operating characteristic curve (AUC). When multiple hyperparameter configurations yielded comparable AUC values, the model with the lower Brier score was selected. Fixed random seeds (1234) were used throughout the analysis to ensure full reproducibility.

**LR**: Logistic regression served as the reference baseline model and was fitted using the default parameter settings. Owing to its deterministic nature and limited hyperparameter complexity, no further tuning was required.

**DT**: A classification decision tree was developed using the rpart package. Tree complexity was regulated by the complexity parameter (cp), which was tuned within the training set. The final model adopted cp = 0.01, while all other parameters were kept at their default values.

**SVM**: Support vector machine classification was performed using the e1071 package with a linear kernel. The regularization parameter was set to C = 1, and probability estimation was enabled to obtain predicted probabilities for model evaluation. Given that linear SVMs are deterministic once the data and parameters are fixed, no additional random seed was necessary.

**LightGBM**: The LightGBM model was implemented for binary classification with the objective function set to binary. The final model comprised 981 boosting trees, selected according to performance within the training dataset. All other hyperparameters were retained at their default values.

**XGBoost**: The XGBoost model was trained using the xgb.train function with a tree-based booster (booster = "gbtree") and a binary logistic objective (objective = "binary:logistic"). Hyperparameters were specified as follows: learning rate (η) = 0.30, maximum tree depth = 2, subsample ratio = 0.70, column subsampling ratio = 0.40, and gamma = 0.001. The maximum number of boosting iterations was set to 1,000. To control overfitting, early stopping was applied based on validation log-loss, with training halted if no improvement was observed for 200 consecutive rounds. The optimal model was achieved at 27 boosting iterations, and this configuration was retained for all subsequent analyses. A fixed random seed was used to ensure reproducibility.

Table S5. Statistical comparison of discrimination and calibration performance among five predictive models in the training and validation datasets

|  | Model | AUC (95% CI) | *p* value | Brier score | Speigelhalter'z | Spiegelhalter'p |
| --- | --- | --- | --- | --- | --- | --- |
| Training | XGBoost (Reference) | 0.829 (0.788-0.870) |  | 0.133 | 0.01 | 0.992 |
|  | LR | 0.731 (0.682-0.780) | 0.003 | 0.161 | 0.000 | 1 |
|  | DT | 0.788 (0.739-0.837) | 0.015 | 0.128 | 0.000 | 1 |
|  | SVM | 0.709 (0.660-0.759) | 0.000 | 0.176 | 0.34 | 0.733 |
|  | LightGBM | 0.786 (0.741-0.831) | 0.006 | 0.145 | -0.16 | 0.874 |
| Validation | XGBoost (Reference) | 0.808 (0.742-0.874) |  | 0.134 | -1.84 | 0.066 |
|  | LR | 0.749 (0.675-0.824) | 0.017 | 0.145 | -1.3 | 0.193 |
|  | DT | 0.746 (0.679-0.814) | 0.025 | 0.178 | -1.92 | 0.055 |
|  | SVM | 0.743 (0.669-0.816) | 0.028 | 0.160 | -0.85 | 0.396 |
|  | LightGBM | 0.721 (0.641-0.800) | 0.002 | 0.155 | -2.34 | 0.019 |

XGBoost, extreme gradient boosting; LightGBM, light gradient boosting machine; SVM, support vector machine; DT, decision tree; LR, logistic regression.

Table S5 presents the statistical comparison of discrimination and calibration among the five models in the training and validation datasets. XGBoost achieved the highest AUC in both datasets (0.829 and 0.808, respectively), with DeLong’s test showing significantly higher discrimination than logistic regression, decision tree, support vector machine, and LightGBM (all p < 0.05). Calibration assessment using the Brier score and Spiegelhalter’s Z test showed that XGBoost yielded the lowest Brier score and no evidence of significant lack of fit in either dataset (p = 0.992 in training; p = 0.066 in validation).

Table S6. Bootstrap-based internal validation results for model discrimination.

| Model | Mean AUC | SD | Median | 95% CI (AUC) |
| --- | --- | --- | --- | --- |
| XGBoost | 0.782 | 0.060 | 0.787 | 0.650–0.888 |
| LightGBM | 0.775 | 0.057 | 0.779 | 0.644–0.869 |
| SVM | 0.736 | 0.066 | 0.738 | 0.599–0.859 |
| DT | 0.667 | 0.076 | 0.670 | 0.500–0.809 |
| LR | 0.749 | 0.037 | 0.753 | 0.667–0.821 |

XGBoost, extreme gradient boosting; LightGBM, light gradient boosting machine; SVM, support vector machine; DT, decision tree; LR, logistic regression.

Table S7. Bootstrap-based internal validation results for model calibration.

| Model | Mean Brier Score | SD | 95% CI (Brier) | Calibration Slope (Mean, 95% CI) | Mean HL *p* value |
| --- | --- | --- | --- | --- | --- |
| XGBoost | 0.145 | 0.021 | 0.105 – 0.188 | 1.11 (0.51–1.80) | 0.245 |
| LightGBM | 0.147 | 0.021 | 0.107 – 0.188 | 1.17 (0.57–1.77) | 0.212 |
| SVM | 0.155 | 0.021 | 0.089 – 0.214 | 1.47 (−0.18–3.12) | 0.169 |
| Decision Tree | 0.169 | 0.026 | 0.119 – 0.218 | 0.59 (0.07–1.24) | 0.138 |
| LR | 0.161 | 0.009 | 0.143 – 0.179 | 1.00 (0.92–1.03) | 0.555 |

XGBoost, extreme gradient boosting; LightGBM, light gradient boosting machine; SVM, support vector machine; DT, decision tree; LR, logistic regression.

Bootstrap-based internal validation confirmed that the XGBoost model demonstrated the most robust predictive performance among all evaluated algorithms. Across 500 bootstrap resamples, XGBoost achieved the highest mean AUC (0.782), indicating superior discrimination ability, and the lowest mean Brier score (0.145), reflecting the most accurate probability calibration. In contrast, LightGBM showed slightly lower discrimination, while logistic regression and the decision tree exhibited reduced stability and calibration accuracy. The calibration slope of XGBoost remained close to 1.0, and Hosmer–Lemeshow tests indicated no evidence of lack of fit, supporting the model’s reliability. These findings demonstrate that XGBoost provides the best balance of discrimination and calibration, and its performance remained stable under extensive bootstrap validation.

Table S8. Confusion matrices of all predictive models evaluated on the validation dataset

| Model | TP | FP | TN | FN | Sensitivity | Specificity | PPV | NPV | F1 |
| --- | --- | --- | --- | --- | --- | --- | --- | --- | --- |
| XGBoost | 37 | 51 | 133 | 14 | 0.726 | 0.723 | 0.421 | 0.905 | 0.532 |
| LR | 26 | 54 | 130 | 25 | 0.510 | 0.707 | 0.325 | 0.839 | 0.397 |
| DT | 16 | 35 | 160 | 24 | 0.400 | 0.821 | 0.314 | 0.870 | 0.352 |
| SVM | 36 | 68 | 116 | 15 | 0.706 | 0.630 | 0.346 | 0.886 | 0.465 |
| LightGBM | 40 | 87 | 97 | 11 | 0.784 | 0.527 | 0.315 | 0.898 | 0.449 |

XGBoost, extreme gradient boosting; LightGBM, light gradient boosting machine; SVM, support vector machine; DT, decision tree; LR, logistic regression; TP, True Positive; FP, False Positive; TN, True Negative; FN, False Negative; PPV, Positive Predictive Value; NPV, Negative Predictive Value;

Figure 1. The potential risk factors were selected using the LASSO regression.


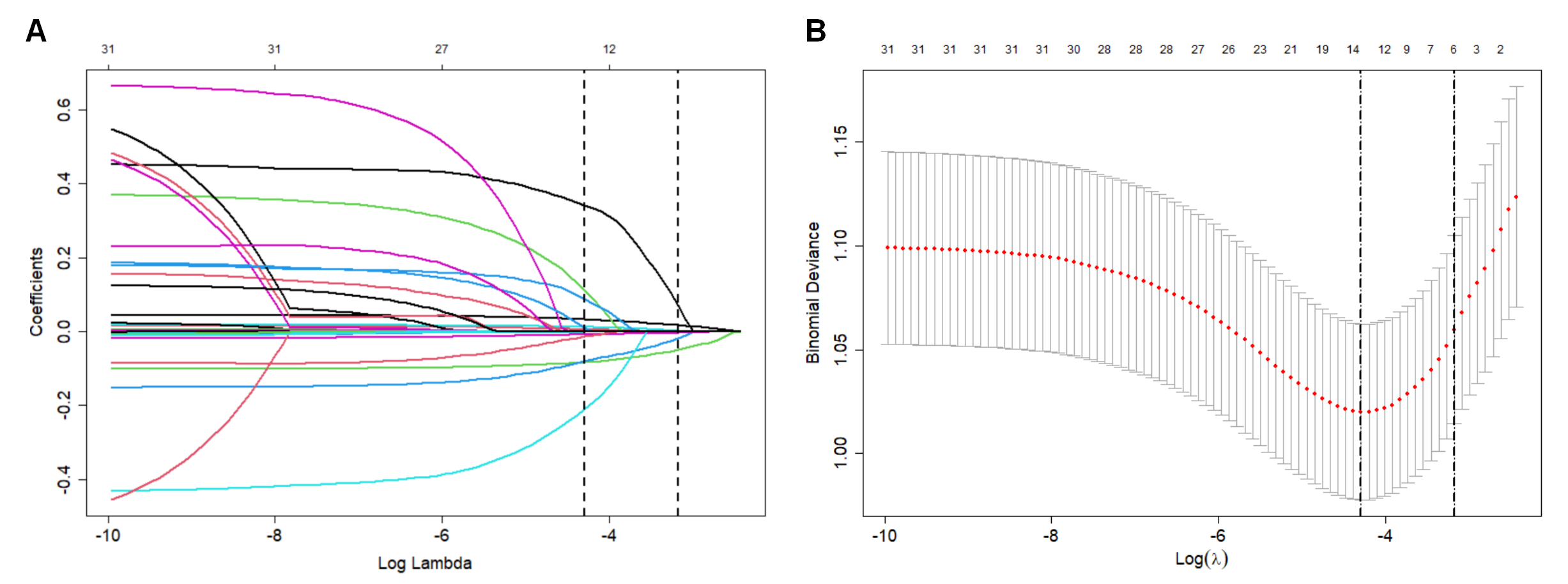


A: Trend graph of variance filter coefficients. Each colour curve represents a trend in the variance coefficient change. B: Graph of cross-validation results. The vertical line on the left side represents λ min, and the vertical line on the right side represents λ 1se. λ min refers to the λ value corresponding to the minimum mean squared error (MSE) among all λ values; λ 1se refers to the λ value corresponding to the simplest and best model obtained after cross-validation within a square difference range of λ min.

**2. Supplementary TRIPOD checklist**

| **Section/Topic Item** | | **Development / evaluation**1 | **Checklist item** | **Reported on page** |
| --- | --- | --- | --- | --- |
| **TITLE** | | | |  |
| *Title* | 1 | D; E | Identify the study as developing or evaluating the performance of a multivariable prediction model, the target population, and the outcome to be predicted | Page 1 |
| **ABSTRACT** | | | | |
| *Abstract* | 2 | D; E | See TRIPOD+AI for Abstracts checklist | Page 2 |
| **INTRODUCTION** | | | | |
| *Background* | 3a | D; E | Explain the healthcare context (including whether diagnostic or prognostic) and rationale for developing or evaluating the prediction model, including references to existing models | Page 3 |
|  | 3b | D; E | Describe the target population and the intended purpose of the prediction model in the context of the care pathway, including its intended users (e.g., healthcare professionals, patients, public) | Page 3 |
|  | 3c | D; E | Describe any known health inequalities between sociodemographic groups | Page 3 |
| *Objectives* | 4 | D; E | Specify the study objectives, including whether the study describes the development or validation of a prediction model (or both) | Page 3 |
| **METHODS** | | | | |
| *Data* | 5a | D; E | Describe the sources of data separately for the development and evaluation datasets (e.g., randomised trial, cohort, routine care, or registry data), the rationale for using these data, and the representativeness of the data | Pages 3-4 |
|  | 5b | D; E | Specify the dates of the collected participant data, including the start and end of participant accrual and, if applicable, the end of follow-up | Pages 3-4 |
| *Participants* | 6a | D; E | Specify key elements of the study setting (e.g., primary care, secondary care, general population), including the number and location of centers | Pages 3-4 |
|  | 6b | D; E | Describe the eligibility criteria for study participants | Pages 3-4 |
|  | 6c | D; E | Give details of any treatments received and how they were handled during model development or evaluation, if relevant | Not applicable |
| *Data preparation* | 7 | D; E | Describe any data pre-processing and quality checking, including whether this was similar across relevant sociodemographic groups | Pages 3-4 |
| *Outcome* | 8a | D; E | Clearly define the outcome that is being predicted and the time horizon, including how and when  assessed, the rationale for choosing this outcome, and whether the method of outcome assessment is consistent across sociodemographic groups | Pages 3-4 |
|  | 8b | D; E | If outcome assessment requires subjective interpretation, describe the qualifications and demographic characteristics of the outcome assessors | Not applicable |
|  | 8c | D; E | Report any actions to blind assessment of the outcome to be predicted | Not applicable |
| *Predictors* | 9a | D | Describe the choice of initial predictors (e.g., literature, previous models, all available predictors) and any pre-selection of predictors before model building | Pages 4-5 |
|  | 9b | D; E | Clearly define all predictors, including how and when they were measured (and any actions to blind assessment of predictors for the outcome and other predictors) | Pages 4-5 |
|  | 9c | D; E | If predictor measurement requires subjective interpretation, describe the qualifications and demographic characteristics of the predictor assessors | Not applicable |
| *Sample* *size* | 10 | D; E | Explain how the study size was arrived at (separately for development and evaluation), and justify that the study size was sufficient to answer the research question. Include details of any sample size  calculation | Pages 4-5 |
| *Missing data* | 11 | D; E | Describe how missing data were handled. Provide reasons for omitting any data | Page 5 |
| *Analytical methods* | 12a | D | Describe how the data were used (e.g., for development and evaluation of model performance) in the analysis, including whether the data were partitioned, considering any sample size requirements | Page 5 |
|  | 12b | D | Depending on the type of model, describe how predictors were handled in the analyses (functional form, rescaling, transformation, or any standardization). | Not applicable |
|  | 12c | D | Specify the type of model, rationale2, all model-building steps, including any hyperparameter tuning, and method for internal validation | Page 5 |
|  | 12d | D; E | Describe if and how any heterogeneity in estimates of model parameter values and model performance was handled and quantified across clusters (e.g., hospitals, countries). See TRIPOD-Cluster foradditional considerations3 | Not applicable |
|  | 12e | D; E | Specify all measures and plots used (and their rationale) to evaluate model performance (e.g., discrimination, calibration, clinical utility) and, if relevant, to compare multiple models | Page 5 |
|  | 12f | E | Describe any model updating (e.g., recalibration) arising from the model evaluation, either overall or for particular sociodemographic groups or settings | Not applicable |
|  | 12g | E | For model evaluation, describe how the model predictions were calculated (e.g., formula, code, object, application programming interface) | Page 5 |
| *Class imbalance* | 13 | D; E | If class imbalance methods were used, state why and how this was done, and any subsequent methods to recalibrate the model or the model predictions | Not applicable |
| *Fairness* | 14 | D; E | Describe any approaches that were used to address model fairness and their rationale | Not applicable |
| *Model output* | 15 | D | Specify the output of the prediction model (e.g., probabilities, classification). Provide details and rationale for any classification and how the thresholds were identified | Page 5 |

| *Training versus evaluation* | 16 | D; E | Identify any differences between the development and evaluation data in a healthcare setting, eligibility criteria, outcome, and predictors | Page 5 |
| --- | --- | --- | --- | --- |
| *Ethical approval* | 17 | D; E | Name the institutional research board or ethics committee that approved the study and describe the participant-informed consent or the ethics committee waiver of informed consent | Page 11 |
| **OPEN SCIENCE** | | | | |
| *Funding* | 18a | D; E | Give the source of funding and the role of the funders for the present study | Page 11 |
| *Conflicts of interest* | 18b | D; E | Declare any conflicts of interest and financial disclosures for all authors | Page 11 |
| *Protocol* | 18c | D; E | Indicate where the study protocol can be accessed or state that a protocol was not prepared | Not prepared |
| *Registration* | 18d | D; E | Provide registration information for the study, including register name and registration number, or state that the study was not registered | Not registered |
| *Data sharing* | 18e | D; E | Provide details of the availability of the study data | Page 11 |
| *Code sharing* | 18f | D; E | Provide details of the availability of the analytical code4 | Page 11 |
| **PATIENT & PUBLIC INVOLVEMENT** | | | | |
| *Patient & Public Involvement* | 19 | D; E | Provide details of any patient and public involvement during the design, conduct, reporting, interpretation, or dissemination of the study or state no involvement. | No involvement |
| **RESULTS** | | | | |
| *Participants* | 20a | D; E | Describe the flow of participants through the study, including the number of participants with and without the outcome and, if applicable, a summary of the follow-up time. A diagram may be helpful. | Fig 1 |
|  | 20b | D; E | Report the characteristics overall and, where applicable, for each data source or setting, including the key dates, key predictors (including demographics), treatments received, sample size, number of outcome events, follow-up time, and amount of missing data. A table may be helpful. Report any differences across key demographic groups. | Page 5 |
|  | 20c | E | For model evaluation, show a comparison with the development data of the distribution of important predictors (demographics, predictors, and outcome). | Table 1 |
| *Model development* | 21 | D; E | Specify the number of participants and outcome events in each analysis (e.g., for model development, hyperparameter tuning, model evaluation) | Table S4 |
| *Model*  *specification* | 22 | D | Provide details of the full prediction model (e.g., formula, code, object, application programming interface) to allow predictions in new individuals and to enable third-party evaluation and implementation, including any restrictions to access or re-use (e.g., freely available, proprietary)5 | Table S4 |
| *Model*  *performance* | 23a | D; E | Report model performance estimates with confidence intervals, including for any key subgroups (e.g., sociodemographic). Consider plots to aid the presentation. | Table 3 |
|  | 23b | D; E | If examined, report results of any heterogeneity in model performance across clusters. See TRIPOD Cluster for additional details3. | Not applicable |
| *Model updating* | 24 | E | Report the results from any model updating, including the updated model and subsequent performance | Not applicable |
| **DISCUSSION** | | | | |
| *Interpretation* | 25 | D; E | Give an overall interpretation of the main results, including issues of fairness in the context of the objectives and previous studies | Page 8 |
| *Limitations* | 26 | D; E | Discuss any limitations of the study (such as a non-representative sample, sample size, overfitting, or missing data) and their effects on any biases, statistical uncertainty, and generalizability | Page 10 |
| *Usability of the*  *model in the*  *context of current care* | 27a | D | Describe how poor quality or unavailable input data (e.g., predictor values) should be assessed and handled when implementing the prediction model | Not applicable |
|  | 27b | D | Specify whether users will be required to interact in the handling of the input data or use of the model and what level of expertise is required of users | Not applicable |
|  | 27c | D; E | Discuss any next steps for future research, with a specific view to the applicability and generalizability of the model | Page 10 |

From: Collins GS, Moons KGM, Dhiman P, et al. *BMJ* 2024;385:e078378. doi:10.1136/bmj-2023-078378
